# Parallel Tubular Channel Angular Pressing (PTCAP) Processing of the Cu-20.7Zn-2Al Tube

**DOI:** 10.3390/ma15041469

**Published:** 2022-02-16

**Authors:** Mohamed Ibrahim Abd El Aal, Elshafey Ahmed Gadallah

**Affiliations:** 1 Mechanical Engineering Department, College of Engineering at Wadi Addawaser, Prince Sattam Bin Abdulaziz University, Wadi Addawaser 18734, Saudi Arabia; eng_mohabdelall@yahoo.com or or; 2 Mechanical Design & Production Department, Faculty of Engineering, Zagazig University, Zagazig 44519, Egypt; 3 Mechanical Production Department, Faculty of Technology & Education, Suez University, Suez 43527, Egypt

**Keywords:** PTCAP, Cu-20.7Zn-2Al tubes, microstructure, tensile properties, hardness, fracture surface, wear resistance

## Abstract

Commercial Al-brass tube was successfully processed by Parallel Tubular Channel Angular Pressing (PTCAP) in 2 passes under an imposed strain of 1.49 per pass. The effect of the number of PTCAP passes on the microstructure and the mechanical properties (hardness, tensile, and wear mass loss) of the Al-brass tubes was fully investigated. The average grain size of the as-received tube decreased to 1.28 μm after up to two passes of PTCAP with a mixture of ultrafine grain (UFG) and coarse grain (CG). The annealed tubes’ tensile strength and Vickers hardness increased by 237.65% and 175.6%, respectively, after two passes. In addition, a ductile fracture occurred with a clear necking. The fracture surface morphology indicated an apparent decrease in dimple size after PTCAP processing, combined with a decrease in ductility. Moreover, the wear mass loss decreased with increasing number of PTCAP passes due to the decrease in the grain size, and the increase of the hardness of the tubes was enhanced after PTCAP processing.

## 1. Introduction

The effect of severe plastic deformation (SPD) on improving the mechanical and physical properties of bulk metal and metal alloy samples has been the motivation of many recent works [1,2,3]. The SPD processing of bulk specimens includes many different processes, especially equal channel angular pressing (ECAP) and high-pressure torsion (HPT) [4,5,6,7,8,9,10,11]. Moreover, the SPD of sheets has been performed by accumulative roll bonding (ARB) [12], equal-channel angular rolling (ECAR) [13], and constrained groove pressing (CGP) [14]. Furthermore, SPD is used in tube specimen processing using spin extrusion (SE) [15,16], high-pressure tube twisting (HPTT) [17], and accumulative spin-bonding (ASB). Moreover, the production of UFG tubes has been carried out using accumulative spin-bonding (ASB) [18], equal-channel angular pressing of tubular (ECAP-tube) [19,20], and tube channel pressing (TCP) [21]. However, these techniques have a high cost and entail great difficulty in setting up the dies, while suffering from the limitation of producing low inhomogeneous microstructures.

Copper and Al-brass tubes are considered essential products for use in a variety of applications. Recently, the demand for Cu and Al-brass tubes with high strength and hardness, as well as superior wear and corrosion resistance, has increased. The production of fine-grain-microstructure Cu and Al-brass tubes using PTCAP has been the motivation of a variety of work [22,23,24,25,26]. Recently, Faraji et al. and M. Mesbah et al. used PTCAP to fabricate ultrafine-grain (UFG) and nonstructural (NS) materials. The advantages of PTCAP have led to an increase in its use in tube processing compared to the Tubular Channel Angular Pressing (TCAP) process [25,26,27,28,29,30,31]. Interestingly, most previous work with the PTCAP of Cu and Al-brass has been performed using die angles of 120° to 150° and pressing rates of 5 to 10 mm/min. Therefore, further investigations on the effect of using a combination of smaller die angles with a slower pressing rate in order to produce a mixture of fine- and coarse-grain Cu and Al-brass tubes with high mechanical properties are still required.

The aims of the present research are as follows. First, to investigate the effect of PTCAP processing using a new die with an angle of 135° on the microstructure evolution of commercial Cu-20.7Zn-2Al tubes. Second, to study the effect of using a smaller PTCAP die angle than those previously used to produce commercial Cu-20.7Zn-2Al tubes with a microstructure consisting of a mixture of fine and coarse grains. Third, to explore the influence of grain refinement after PTCAP of the Cu-20.7Zn-2Al tubes on their mechanical properties.

## 2. Materials and Methods

### 2.1. Mechanical and Microstructure Properties

**Figure 1:**
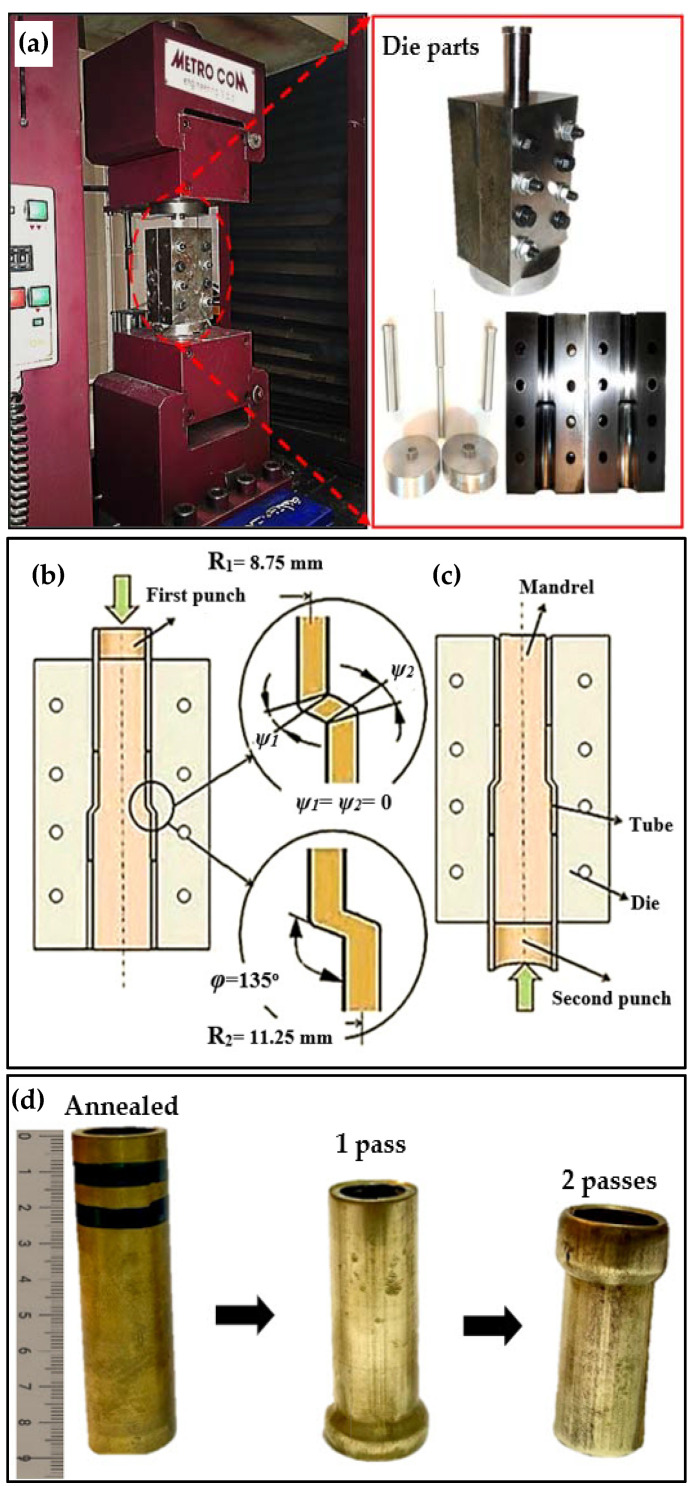
Schematic of the PTCAP process: (**a**) experimental setup, (**b**) first half cycle, (**c**) second half cycle, and (**d**) unprocessed and PTCAP processed specimens (after 1 and 2 passes).

Cu-20.7Zn-2Al tubes, with compositions listed in Table 1 and with an outer diameter of 20 mm, a thickness of 2.5 mm, and a length of 80 mm, were used in the present work. For one hour, the tubes were annealed at 700 °C in a vacuum furnace under argon gas. The different parts of the PTCAP die are shown in Figure 1a. The die geometry parameters include channel angles φ_1_ = φ_2_ of 135°, angles of curvature *ψ*_1_ = *ψ*_2_ of 0°, and tube thickness *K* = *R*_2_ − *R*_1_ = 2.5 mm, where R_2_ and R_1_ are the outer and inner tube radii (Figure 1b,c). The PTCAP die parameters used in the current study were selected on the basis of a previous finite element study [32]. Low die angles are recommended in order to introduce a higher deformation homogeneity and finer grain. Therefore, an angle of 135° was chosen to achieve ultra-fine grains, low load, and high strain homogeneity. Molybdenum disulfide (MoS2) was used as a lubricant during the PTCAP of the Cu-20.7Zn-2Al tubes [33]. The Al-brass tubes were pressed in up to two passes at room temperature (Figure 1d) at a speed of 2 mm/min. The PTCAP process consists of two half cycles (Figure 1). The first pass began with placing the tube between the mandrel and the die. A cylindrical punch with exactly the same dimensions as the tube was used to extrude the tube from the smaller diameter of 20 mm with a thickness of 2.5 mm to the larger diameter of 25 mm and a thickness of 2.5 mm (Figure 1b). The second pass started with the inversion of the die and the re-extrusion of the tube in the channel using the second punch from the larger diameter, and the tube diameter returned to its original size (Figure 1c). The imposed strain in each pass was 1.49, as calculated using Equation (1) [28] on the basis of the different parameters of the PTCAP die:

(1) ε¯PTCAP=2N{∑i=12[2cot(φi2+ψi2)+ψicosec(φi2+ψi2)3]+23lnR2R1 } 

where the *N*, *φ*, *ψ*, *R*_2_, *R*_1_ parameters are the number of passes, die channel angle, die angle of curvature, and tube outer and inner radius, respectively, as shown in Figure 1b,c. The maximum imposed strain through the PTCAP after 2 passes before tube fracture was 2.98.

The annealed and PTCAPed tube microstructures and the average grain size [34] were examined and obtained using an Olympus optical microscope (OM) model (BX41M-LED, Center Valley, PA, USA). Mechanical polishing, grinding, and etching were carried out for specimens before and after the PTCAP process. The etching was carried out using a solution of 5g FeCl_3_, 100 mL ethanol, 5 mL HCL, for 10–40 s [35].

**Figure 2:**
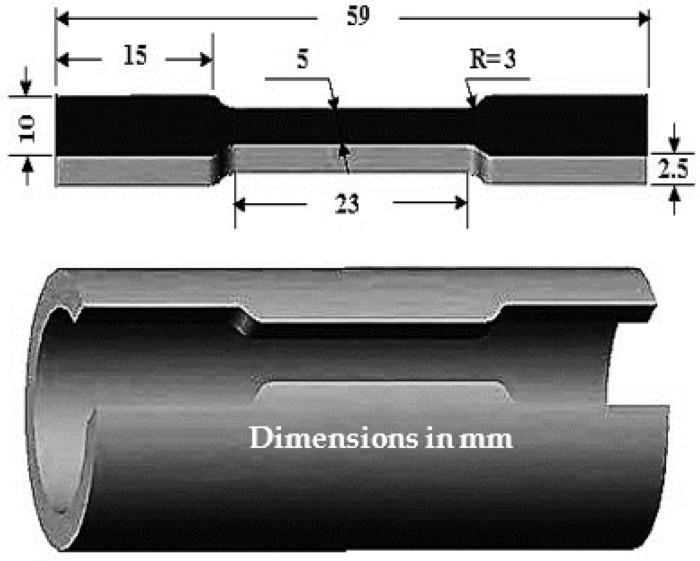
Schematic of the dimensions of the tensile test specimen.

Dog bone tensile specimens with dimensions as shown in Figure 2 were punched from the tubes as shown in Figure 2. Tensile testing was conducted up to failure at room temperature using a universal testing machine—Instron model (4208-002)—operated at a constant strain rate of 3 × 10^−3^ s^−1^. The test was repeated three times for each condition to obtain more reliable results. The tensile sample’s fracture surface morphology was investigated using an SEM model FEI INSPECT-S50 (FEI, Austin, TX, USA) at 20 kV.

The hardness of the Al-brass tubes was measured on their faces for both the inner and the outer radii. The tube’s surface was carefully prepared by grinding followed by polishing. The hardness was measured on the basis of Vickers hardness under a load of 500 g and a dwell time of 15 s, along six different radii for each sample with an interspacing of 0.5 mm between the measurement locations. The average hardness value at the six different readings in the same position for each radius was obtained and drawn to show the hardness distribution in each case. On the other hand, the average value of all measurements is presented as the overall hardness value for each case. The standard deviation of the hardness measurements was calculated to assess the deformation inhomogeneity index (*H_index_*) according to Equation (2) [36,37].

(2) $$\;H_{index}=\sqrt{\frac{\sum {\left(H_{i}-H_{av}\right)}^{2}}{N_{h}}}\;$$

where *H_i_, H_av_*, and *N_h_* denote the hardness value at each point of measurement, the average hardness of all of the points of measurement, and the total number of measurements, respectively.

### 2.2. Wear Properties

**Figure 3:**
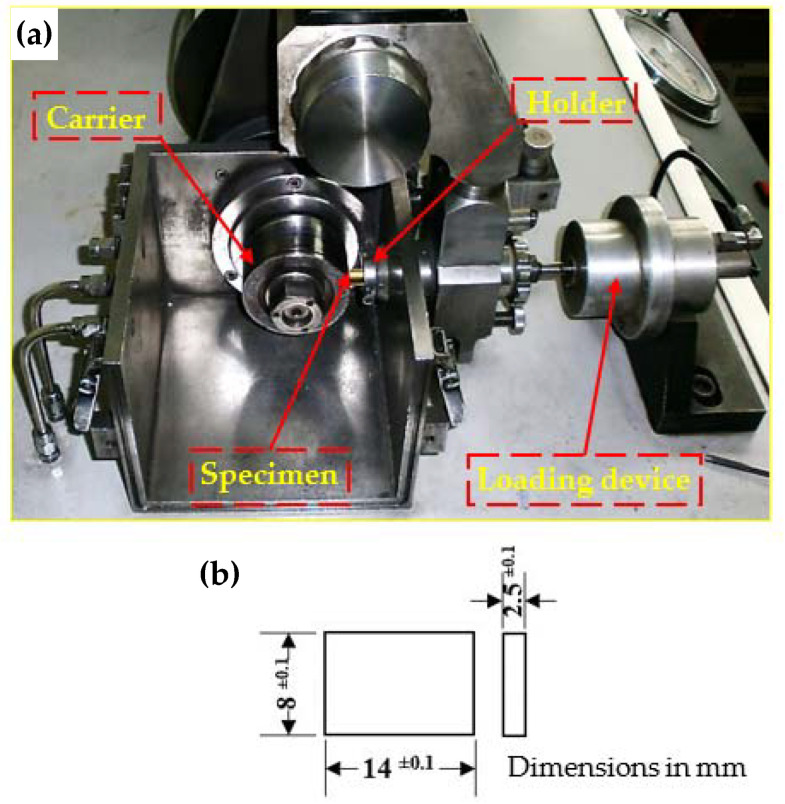
(**a**) Block-on-ring test rig (ASTM) model TRIBOMETER, and (**b**) wear test specimen.

Dry sliding wear tests were carried out using a TNO Tribometer (Irvine, CA, USA) block-on-ring wear tester, as shown in Figure 3a. Block-shaped specimens with dimensions of 2.5 × 8 × 14 mm^3^, as shown in Figure 3b, were cut from the tubes before and after PTCAP using wire EDM. Wear specimens were ground and polished carefully, then cleaned in acetone bath in an ultrasonic device. The wear test was performed against a steel ring 73 mm in diameter and with a hardness of 63 Rc. The wear test was carried out at room temperature (20 ± 5 °C) and 50 ± 5% humidity with sliding distances of 1375.2 m and 2062.8 m under applied loads of 30, 40, and 50 N. The sliding distance can be defined as the linear speed times the duration of the wear test. In the present work, two different speeds—68.76 and 45.84 m/min—were used for 30 min, resulting in sliding distances of 1375.2 and 2062.8 m, respectively. The selected wear test parameters, the sliding distances, and the applied loads were selected on the basis of a comprehensive study of the wear of Cu, Cu alloys, and composites when severely deformed [38,39]. The wear mass loss (g) was determined using a digital balance device with a sensitivity of 10^−6^ kg.

## 3. Results

### 3.1. Microstructure Characterizations

**Figure 4:**
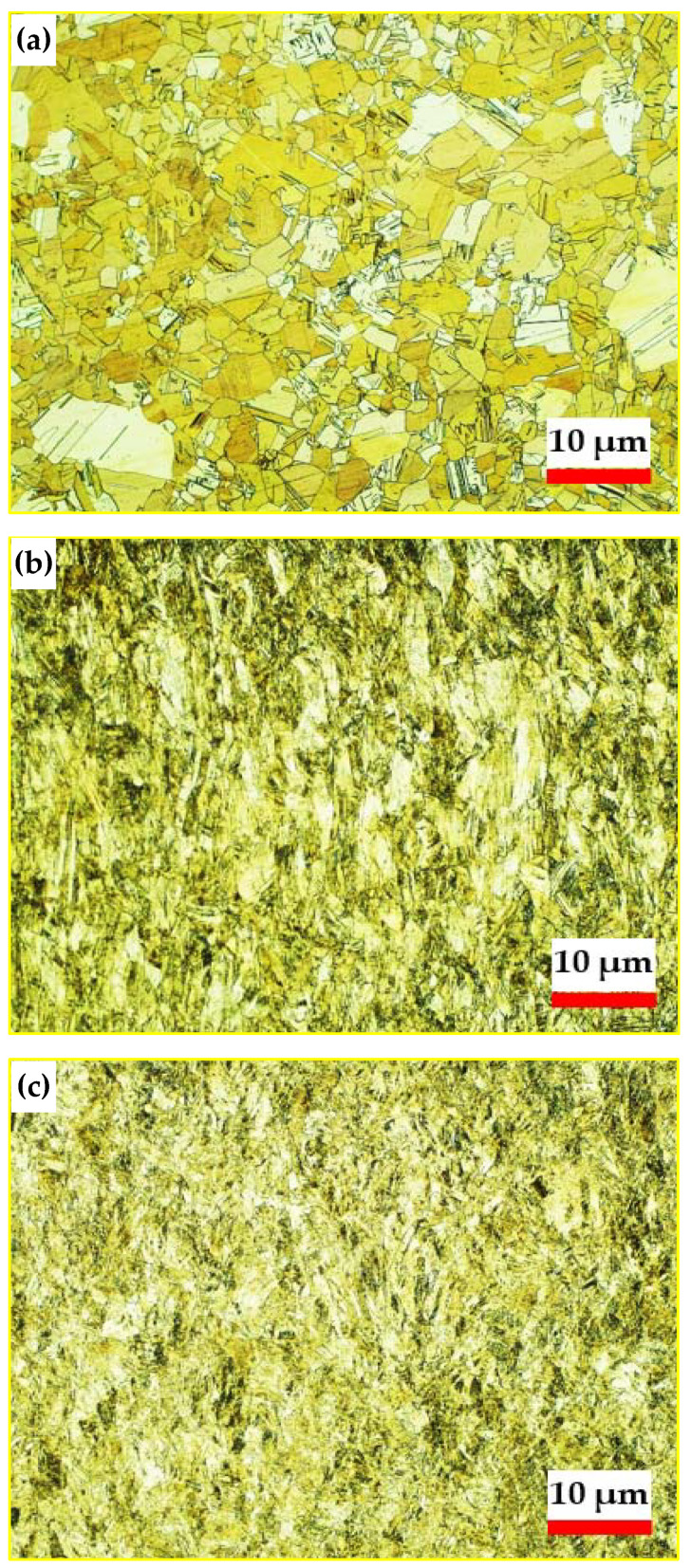
Optical microstructure of the Al-Brass tube: (**a**) annealed, and (**b**) after one pass of PTCAP processing, and (**c**) after the second pass.

**Figure 5:**
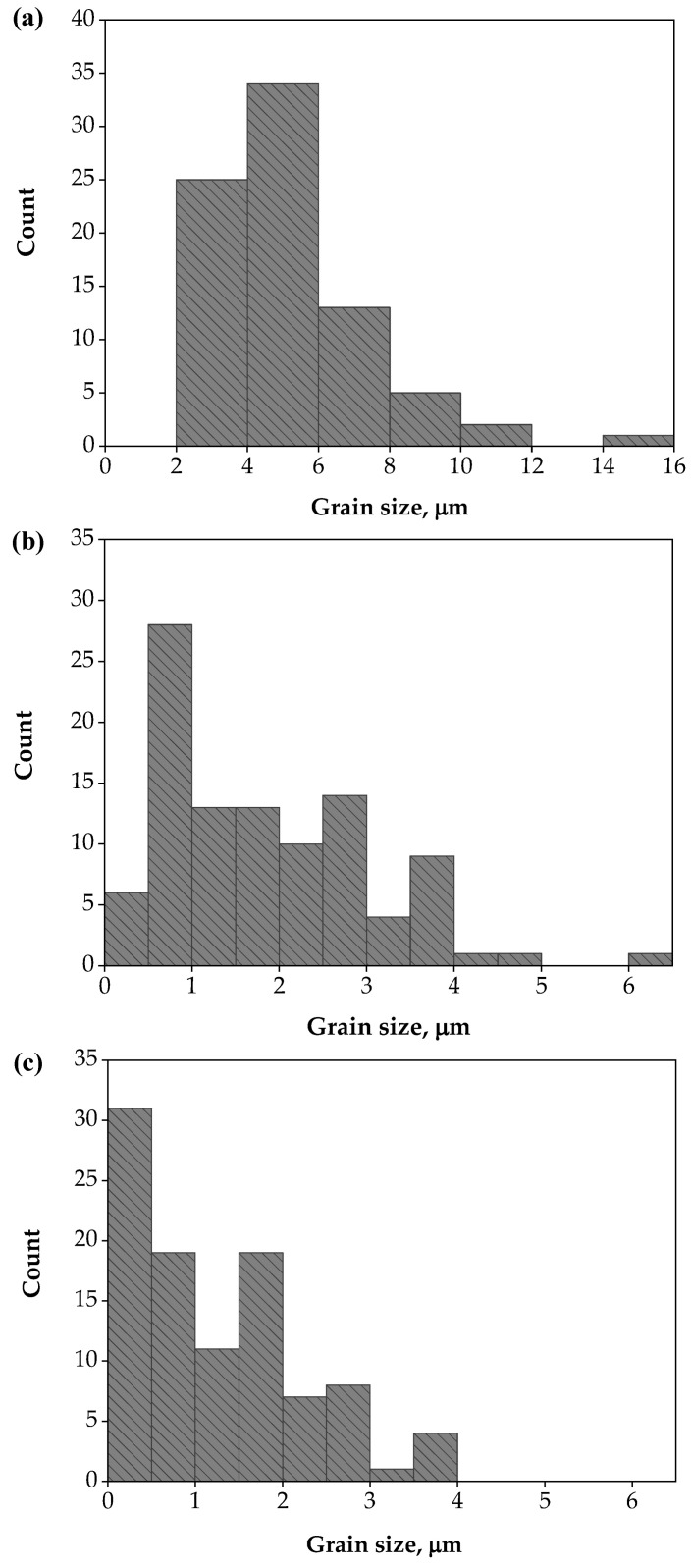
Grain size distribution of (**a**) unprocessed, and (**b**) after the first pass of PTCAP processing, and (**c**) after the second pass.

The annealed Al-brass tube microstructure showed grain size homogeneity, with a mean grain size of 5.07 µm, as shown in Figure 4a. The Al-brass tube microstructure was refined, and the mean grain size decreased to 1.86 and 1.28 µm after the first and second passes, as indicated in Figure 4b,c. Moreover, the Al-brass grains achieved greater refinement due to their hardness, while their brittleness was confirmed, as they showed limited deformation that was only achieved after the second pass, as previously noted in various studies [26,28]. The grain size distributions, shown in Figure 5a–c, indicate a decrease in grain size range following PTCAP processing. The grain size range decreased from 0 < d ≤ 16 µm in the case of the annealed tube to 0 < d ≤ 6 µm and 0 < d ≤ 4 µm one and two passes of PTCAP, respectively. Furthermore, the grain size distribution became more homogenous, contributing to an increase in the mechanical properties, as will be discussed in the following sections. Moreover, the percentage of grains with UFG grain size (grain size less than 1 µm) increased to 33% and 52% after one and two passes of PTCAP, respectively, confirming the effect of the accumulated shear strain during PTCAP in the grain refinement of Al-brass tubes. Interestingly, it was observed that the microstructure of the Al-brass tubes after PTCAP processing was a mixture of micro and UFG grain sizes, contributing to improved tensile strength and hardness, while conserving a reasonable degree of ductility, as previously noted in different severely deformed materials [40,41]. This will be further discussed in the tensile test results.

### 3.2. Mechanical Properties

#### 3.2.1. Tensile Properties and Fracture

**Figure 6:**
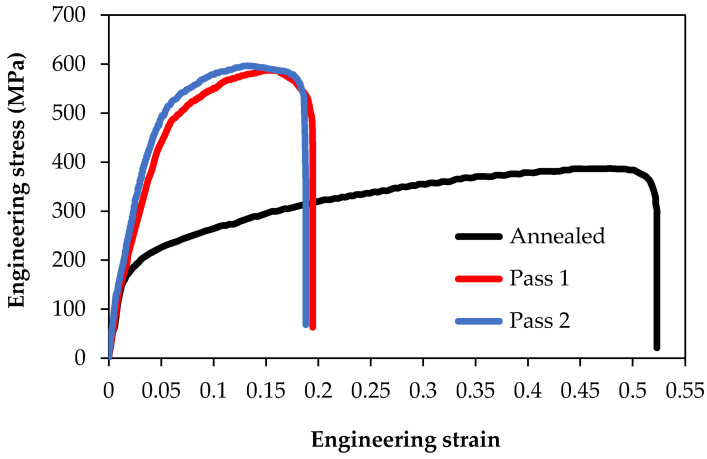
Engineering stress–strain curve for Al-brass tubes of unprocessed and PTCAP-processed specimens.

**Figure 7:**
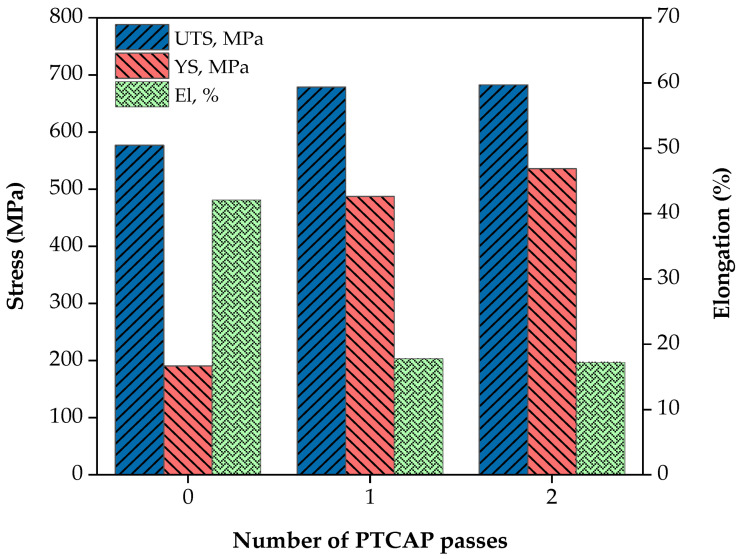
Tensile properties of Al-brass tubes before and after PTCAP processing.

The engineering stress–strain curves and the influence of the PTCAP process on the tensile strength and ductility of the Al-brass tubes are shown in Figure 6. The strength of the PTCAP samples was higher than that of the annealed one (Figure 6 and Figure 7). It was observed that the yield strength and ultimate tensile strength (UTS) of the Al-brass tube was enhanced from 117.94 and 387.13 MPa in the annealed state to 286.89 and 587.28 MPa, and 398.22 and 596.45 MPa after the one and two passes, respectively. Therefore, the yield strength and UTS increased by 143.25–51.71% and 237.65–54.07% after the one and two passes of processing compared to the annealed tube yield strength and UTS. The increase in the strength of the Al-brass tubes can be explained by the grain refinement occurring after PTCAP according to the Hall–Petch relationship [42,43], as indicated in Equation (3).

(3)  σy=σ0+ky D−12 

where *σ_y_*, *σ*_0_, *k*_y_, and *D* are the yield strength, friction strength, yield constant, and grain size. Moreover, the increase of dislocation density contributes to the increase in strength, as previously noted after the PTCAP of Al-brass tubes [2,44,45,46,47,48]. However, recently, different factors can also be considered in explaining the increase in the yield strength of severely deformed materials. The increase in strength can be attributed to grain refinement, grain boundary strengthening, and dislocation strengthening during the deformation processes. The yield strength for polycrystalline metal alloys is reported using the general strength model, modified by Qiao et al. as follows [49]:

(4) $$\sigma_{y}=\sigma_{0}+\Delta \sigma_{gb}+M\tau_{d}\;$$

where *σ_y_*, *σ*_0_, Δ*σ_gb_*, *M* and *τ_d_* are the yield strength, friction strength, strengthening due to the presence of grain or sub-grain boundaries, Taylor factor, and strengthening due to dislocations, respectively. The grain boundary strengthening component, which consists of sub-grain and grain boundary strengthening, Δ*σgb*, can be calculated as presented in Equation (5).

(5) $$\Delta \sigma_{gb}=\alpha_{2}Gb\;\left[\lambda \;f_{sub}\left(\frac{1}{\delta }\right)+\left(1-f_{sub}\right)\left(\frac{1}{D}\right)\;\right]\;$$

where *α*_2_, *G*, *b*, *λ*, *f_sub_*, *δ*, *D* are a constant, the shear modulus, Burgers vector, the ratio of sub-grain boundary strengthening contribution to grain boundary strengthening contribution [36,50,51], the fraction of LAGB, sub-grain size, and grain size, respectively. Moreover, *τ_d_* is the strengthening resulting from dislocations, which can be expressed as Equation (6) by considering the average dislocation density [52]:

(6) $$\tau_{d}=\alpha_{1}Gb\sqrt{\rho }\;\;$$

where *α*_1_ and *ρ* are a constant and average dislocation density, respectively. The current study was concerned with the experimental part of the PTCAP of Al-brass tubes. However, further work on the determination of the grain boundary effect and dislocation density and the theoretical calculation of the tube strength is underway.

Figure 7 shows that the elongation percentage decreased from 52.31% for the annealed specimen to 19.46% and 18.80% after one and two passes, respectively. Compared to previous works, PTCAP using a die with the parameters selected in the current study conserved a reasonable level of ductility while increasing the strength. The microstructures of the PTCAP tubes with a mixture of UFG and micro grain sizes contribute to the conservation of a high degree of ductility with reasonably high strength. The formation of such a microstructure can be explained by the difference in the dislocation density through the deformed material due to the non-homogenized deformation during SPD processes. As a result, localized deformation and dislocation activities occur, and the grain size decreases to nano or UFG sizes in regions with high amounts of dislocation activities. On the other hand, coarse grains remain in regions with low dislocation activity [36,53,54].

**Figure 8:**
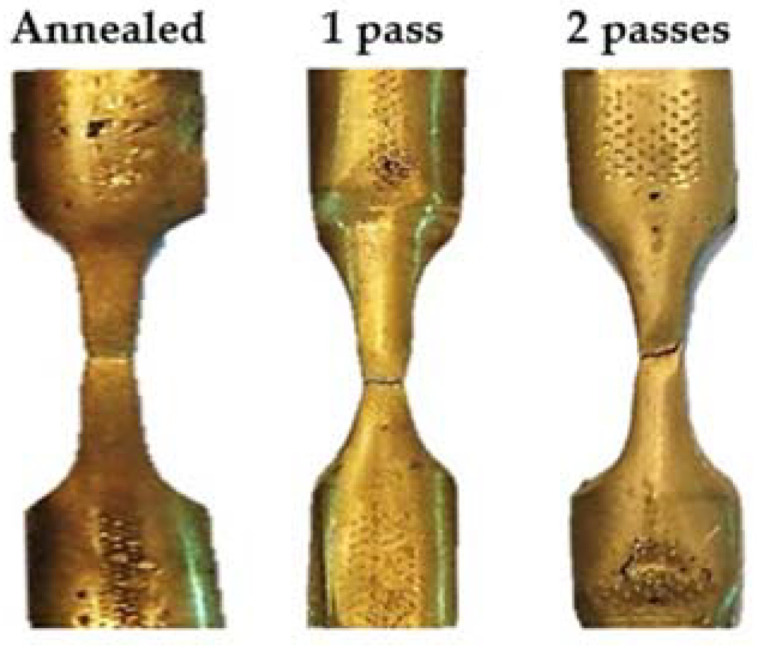
Tensile specimen fracture mode before and after PTCAP processing.

Figure 8 shows the fracture mode of the Al-brass tube before and after PTCAP. The fracture mode for all cases was a ductile mode with an obvious cup and cone fracture mode. However, the cross-section area of the fracture surface decreased with increasing number of PTCAP passes, and transformed into a shear fracture at an angle of 90° with the tensile axis. This observation is in agreement with the tensile results, as the elongation decreased with increasing number of PTCAP passes, as previously noted [29,55].

**Figure 9:**
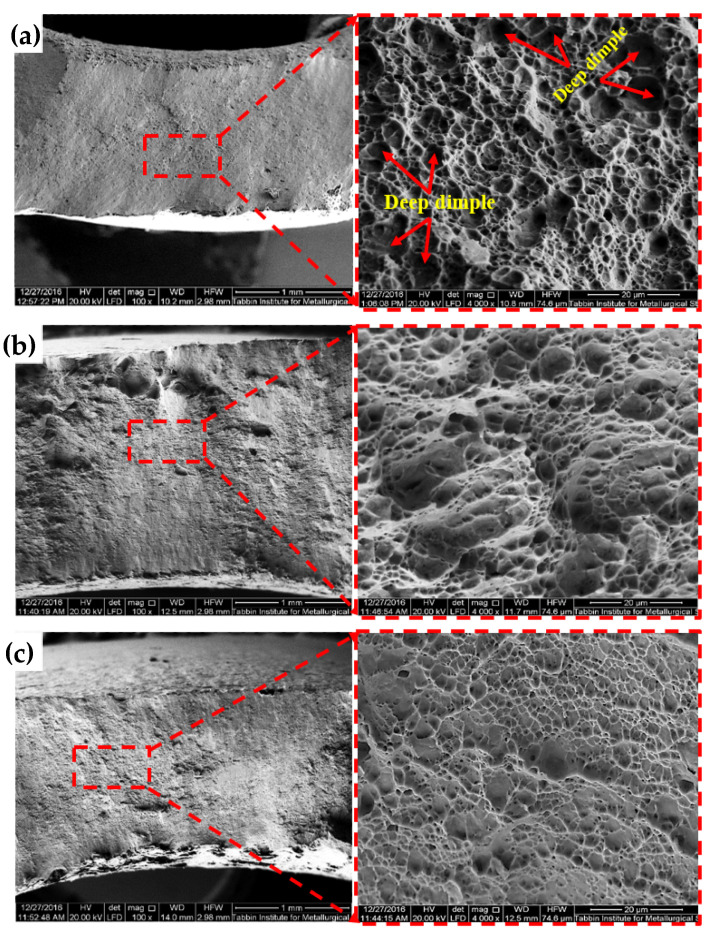
SEM micrographs for the fracture surfaces of Al-brass tube before and after PTCAP processing: (**a**) no passes, (**b**) one pass, and (**c**) two passes.

The fracture surface morphology of the annealed Al-brass tube had large deep dimples (Figure 9a). The fracture surface was considered to consist of ductile fracture after the PTCAP of the Al-brass tube. The size of the dimples of the different specimens decreased after the first and second passes (Figure 9b,c). Similar observations of decreases in dimple size have been noted after SPD processing of various materials [36,41,56]. However, larger deep dimples were noted in the present work compared to those of Al-brass specimens deformed using different processes [47,48,57,58,59]. In other words, it can be noted that due to the higher degree of ductility obtained in the present work, which is related to the presence a microstructure comprising a mixture of UFG and coarse grains, large dimples were still noted.

#### 3.2.2. Hardness Results

**Figure 10:**
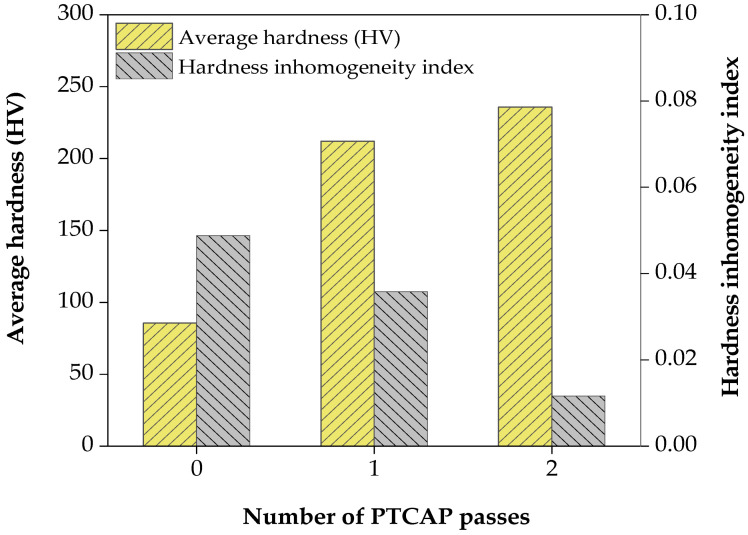
Average hardness and hardness inhomogeneity index of the Al-brass tubes before and after PTCAP passes.

Figure 10 shows that the hardness of the Al-brass tube increased obviously from 85.55 HV to 235.75 HV after the second pass with a percent increase of 175.57% relative to the annealed specimen. The increase of the Al-brass tube hardness was due to a decrease in grain size. The hardness of the Al-brass tube processed with a die angle of 135° and a pressing rate of 2 mm/min was equal to or higher than that of the PTCAP Al-brass tubes presented in [46,47]. Furthermore, the observations of increased hardness in the Al-brass tubes were in agreement with the tensile results and the microstructure observations, where grain refinement contributes to increased hardness according to the Hall–Petch relationship [42,43].

**Figure 11:**
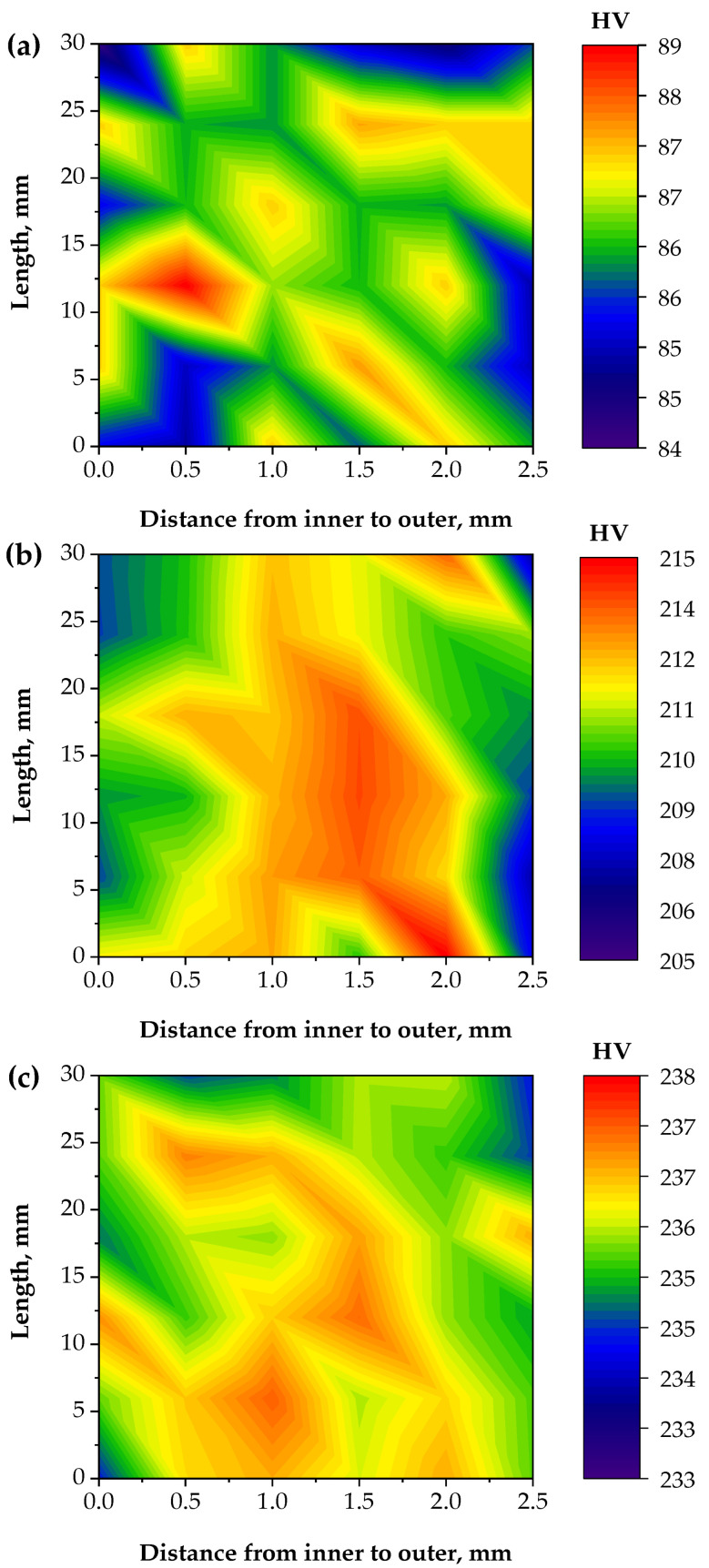
Hardness distribution map of the Al-brass tube along the tube thickness cross-section with distance from the inner to the outer: (**a**) annealed, (**b**) after the first pass, and (**c**) after the second pass of PTCAP processing.

Hardness distribution maps through the tube thickness for the Al-brass tube before and after PTCAP are shown in Figure 11. The Al-brass tube before and after the PTCAP had a homogenous hardness distribution through their thickness. The difference between the maximum and minimum hardness values across the different samples did not increase above 5 HV in all cases, proving the homogenized distribution of the hardness through the tube thickness. Therefore, the PTCAP process produced homogenous tubes with a higher degree of homogeneity than the TCAP and ECAPed specimens, which suffer from the formation of a corner gap, as noted in various materials [22,60,61].

### 3.3. Wear Mass Loss

**Figure 12:**
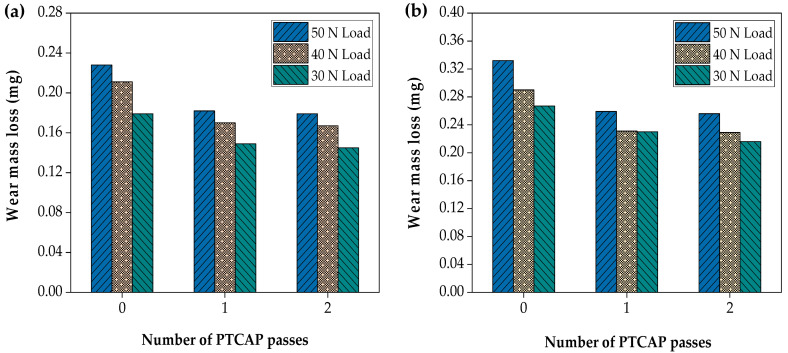
Effect of the number of passes and applied load on the wear mass loss of the Al-brass tube under sliding distance of (**a**) 1375.2 m and (**b**) 2062.8 m.

The influence of the load and distance on the wear mass loss of the Al-brass tube before and after the PTCAP processing is shown in Figure 12. The wear mass loss increased with increases in both the load and distance in all cases. The applied load increased the wear mass loss by 16.19–21.78%, 16–21.58%, and 16.10–22.91%, under loads of 30, 40, and 50 N, respectively, in the case of the Al-brass after one and two passes under different sliding distances (Figure 12a). Moreover, the distance increased from 1375.2 m to 2062.8 m, leading to increases in wear mass loss by 15.62–19.04%, 16.89–22.92%, and 21.94–26.85%, under loads of 30, 40, and 50 N, respectively, in the case of the Al-brass after the first and second passes (Figure 12b). Therefore, the mass loss increased with increases in both the load and the distance and decreased with increasing number of PTCAP passes [59,62]. Moreover, the Al-brass tube before and after the PTCAP had narrower and shallower scratches than the Cu tubes. These observations confirm the improved wear resistance of the PTCAP Al-brass tube compared to the annealed tube.

## 4. Conclusions

In the current research, the following conclusions can be drawn:

1. PTCAP using a die with a channel angle of 135°, a curvature angle of 0°, and a pressing rate of 2 mm/min was successfully performed on Al-brass tube in up to two passes.
2. The average grain size decreased from 5.02 μm before PTCAP to 1.28 μm after two passes of PTCAP processing.
3. The yield strength, UTS, and hardness increased by 237.65%, 54.07%, and 175.6%, respectively, after two passes of PTCAP. In addition, there was about a 64.06% reduction in elongation.
4. Ductile fracture with a clear necking zone and deep dimples was observed in the annealed specimen, while the size and depth of the dimples decreased after PTCAP because of the grain refinement.
5. The wear mass loss decreased with increasing number of PTCAP passes due to the decrease in the grain size and increase in the dislocation density, as well as the increase in hardness.

## Figures and Tables

**Table 1:** Chemical composition of the Al-Brass tube alloy (in wt.%).

Zn	Al	Sn	Pb	Fe	Ni	Mn	Si	P	Be	Mg	As	Cr	S	Co	Cu
20.70	2.00	0.008	0.009	0.013	0.011	0.005	0.015	0.004	0.014	0.00	0.012	0.00	0.00	0.00	Bal.

## Data Availability

The data presented in this study are available on request from the corresponding author. The data are not publicly available due to the extremely large size.
